# Climate counts: a multilevel validation of the EssenCES in Romanian correctional settings

**DOI:** 10.3389/fpsyg.2025.1666516

**Published:** 2025-09-23

**Authors:** Roxana Andreea Toma, Raluca Maria Răducan, Marius Lupsa Matichescu, Emanuel Ionuț Andelin, Rainer Banse

**Affiliations:** ^1^Department of Psychology, West University of Timișoara, Timișoara, Romania; ^2^Department of Sociology, West University of Timișoara, Timișoara, Romania; ^3^“Constantin Brâncoveanu” National Penitentiary Police School - Arad Section, Arad, Romania; ^4^Department of Psychology, Rheinische Friedrich-Wilhelms-Universität Bonn, Bonn, Germany

**Keywords:** prison social climate, EssenCES, therapeutic hold, experienced safety, patient’s/inmate’s cohesion and mutual support, custody regime, forensic hospital

## Abstract

**Introduction:**

The social climate in prisons is associated with better mental health and increased participation in activities.

**Method:**

In a validation study, we examined the predictive validity of the EssenCES for the Romanian prison system. The sample included 566 inmates and patients as well as 175 staff members, 741 in total.

**Results:**

The Romanian version of EssenCES with the three scales Patients’/Inmates’ Cohesion and Mutual Support, Experienced Safety and Therapeutic Hold showed good psychometric properties for prisons and forensic hospitals. Inmates and staff rated the prison climate slightly differently, with staff having a more positive view of therapeutic support than inmates, and inmates having a more positive view of the security on the ward. Staff in semi-open and maximum security regimes reported higher Patient Cohesion scores than inmates in regular prisons, while staff in forensic hospitals reported lower Patient Cohesion scores than inmates in the same settings.

**Discussion:**

Taken together, the findings demonstrate the utility of multilevel and multivariate approaches in clarifying how regime and role shape prison climate, informing practice across forensic and correctional settings.

## Introduction

Violence prevention has become an important public health concern, particularly since 2002 when the World Health Report identified violence as a “global public health problem” (Krug et al., 2002). There is, therefore, a public health need to improve the institutional efforts of offender rehabilitation. The rehabilitation process takes place mainly within the walls of prisons and forensic psychiatric hospitals. Although there are constant efforts to improve the programs and interventions designed to reduce the risk of recidivism, there are still many open questions concerning which measures are conducive to reduce recidivism, and what are the boundary conditions that need to be met for an effective implementation of interventions.

In addition to the specific interventions for inmates and patients, specific aspects of the custodial setting, such as the prison climate, became a major research interest (Schalast and Laan, 2017). A recent and comprehensive definition of the social climate in custodial settings includes “elements of the environment, social culture, interpersonal interactions and relationships that are distinctive to the organization as perceived by those who live and work there” (Bennett and Shuker, 2018, p. 46). It includes the social, emotional, organizational, and physical characteristics of a correctional institution (Ross et al., 2011) as they are perceived by inmates and staff (Tonkin, 2016).

Moos (1968) is credited to have coined the term prison social climate. Schalast and Groenewald (2009) have stated that “the physical, social and emotional conditions of an institutional setting interact in a specific way to create a condition that can be called “social climate or atmosphere,” which over time can influence the mood, behavior and self-concept of the people involved” (Schalast and Laan, 2017, p. 167). The idea of the prison social climate implies the expectation that a proper understanding and management of the social climate in prisons can have beneficial effects on inmates. A high level of cohesion and therapeutic support is expected to open up opportunities for change that facilitate the personal growth of inmates and the adoption of prosocial values. In a systematic review of the relationship between perceived social climate and aggression, Robinson et al. (2018) reported that services fostering positive social climates—defined by safety, cohesion, and supportive environments for staff and patients—consistently exhibited lower aggression levels. More recently, a meta-analysis by Eltink et al. (2024) found that prioritizing safety in residential facilities was associated with reductions in antisocial behavior. Collectively, these findings highlight the need to promote therapeutic environments and deliver evidence-based, rehabilitation-oriented interventions tailored to client needs.

Since the 1960s, several psychological measures have been developed to assess the prison climate, including the Correctional Institutions Environment Scale (Moos, 1975), the Community-Oriented Programs Environment Oriented Scale (Moos, 1974; Moos et al., 1990), the Ward Atmosphere Scale (WAS, Moos, 1996; Moos and Houts, 1968), the Essen Climate Evaluation Schema (EssenCES; Schalast et al., 2008), and the Prison Climate Questionnaire (Bosma et al., 2020).

One important aspect of the concept is the social or therapeutic climate of the prison or forensic psychiatric hospital facility. One often used operationalization of the prison climate is the EssenCES (Cunha et al., 2023). The scale, developed by N. Schalast, assesses key facets of the work environment across three dimensions: therapeutic hold (support for patients’ therapeutic needs), experienced safety (perceived tension and risk of aggression), and patients’ cohesion and mutual support (mutual support characteristic of therapeutic communities; Howells et al., 2009, p. 311). In addition to good psychometric properties (Tonkin et al., 2012), the EssenCES is available in 14 languages (German, English, French, Swedish, Spanish, Portuguese, Polish, Norwegian, Dutch, Korean, Japanese, Italian, Finnish, and Danish) and has been shown to have very good cross-cultural validity. The scales also showed satisfactory reliability in a pilot study in medium and high-security prisons in the US (Williams et al., 2019). Minor linguistic modifications were proposed for the items, predominantly for particular categories such as inmates with disabilities, as mentioned above.

## The present study

We conducted a validation study for the EssenCES in the Romanian prison system for correctional facilities of different security levels and forensic hospitals, similar to previous studies for inmates in prison (Schalast et al., 2008; Tonkin et al., 2012) and patients of forensic psychiatric hospitals (de Vogel and de Ruiter, 2004).

The EssenCES was selected as the primary instrument for assessing prison climate due to its strong empirical support (Tonkin et al., 2012). It comprises three subscales: therapeutic hold (TH), patient’s/inmate’s cohesion (PC) and experienced safety (ES). TH reflects the quality of therapeutic relationships, a key predictor of treatment outcomes (Budman et al., 1993; Luborsky et al., 1985; Martin et al., 2000). PC assesses peer support and group cohesion, aligning with the therapeutic community model (Rapoport, 1960), which is strongly associated with treatment outcome (Beech and Fordham, 1997; Budman et al., 1993; Yalom, 1985). ES captures perceptions of safety, a fundamental human need in environments where aggression may be present (Schalast and Tonkin, 2016).

The EssenCES has demonstrated robustness even in forensic populations with intellectual disabilities, outperforming other measures despite recommended adaptations to language and Likert scales (Chester et al., 2015; Robinson and Craig, 2019). Patients in this group often struggle with abstract language, double negatives, and complex phrasing, and many find Likert scales difficult to interpret. To improve comprehension, the use of pictorial aids has been recommended (Chester et al., 2015).

So far, the EssenCES has not been used in Romanian prisons. In 2022, in Romania, there were approximately 23,000 persons in custody of the Romanian National Prison Administration (ANP) (World Prison Brief). ANP is managing 40 open, semi-open and high-security prisons, four juvenile delinquency educative facilities and six forensic psychiatric hospitals.

Given its impact on rehabilitation outcomes and recidivism prevention, assessing the social climate in prisons and forensic hospitals—including in Romania—is essential. Such measurement tools enable timely management interventions to enhance inmate and patient rehabilitation. Moreover, a positive work environment benefits staff well-being, indirectly supporting rehabilitation efforts. Evaluating social climate aligns effective prison management with promoting personal growth and social justice (Bennett and Shuker, 2018).

This study included three prison regimes—high-security, semi-open, and forensic hospitals—each represented by two facilities. Data were collected from both staff and inmates to examine similarities and differences in their perceptions of prison social climate. The current assessment serves as Time 1 in a planned longitudinal study on the effects of prison climate and risk factors on recidivism.

The primary goals of this study were to validate the EssenCES scale for measuring prison social climate in Romanian forensic prisons and hospitals and to compare these findings with previous large-scale validation studies conducted in Germany, the United Kingdom, and Australia. While the scale has been translated into several languages, these countries represent the main contexts where extensive validation research has been published.

We hypothesized that the EssenCES’s three-factor structure—patient’s/inmate’s cohesion (PC), experienced safety (ES), and therapeutic hold (TH)—would be replicated across prison regimes and forensic hospitals, consistent with findings from Germany, the United Kingdom, and Australia. Additionally, perceived aggression was expected to correlate positively with prison security level. By including three facility types (semi-open prison, high-security prison, and forensic hospital) and two respondent groups (staff and inmates/patients), we aimed to examine in depth more prison climate facets. We anticipated that the items of the three-factor structure model would have the same meanings in both respondent groups (inmates and staff members) and both facility types (prisons and forensic hospitals). We expected significant differences between the two respondent groups regarding EssenCES subscales scores (means). Specifically referring to security level, TH and ES levels were expected to be higher in forensic hospitals. PC was hypothesized to be greater in semi-open prisons and forensic hospitals than in high-security facilities.

## Method

The West University of Timisoara Ethics Committee approved the study (UVT no. 29733/09.05.2023), and the Romanian National Authority of Penitentiaries authorized access to persons who committed (PCO) offenses files, ensuring anonymity and confidentiality (ANP no. 41617/06.09.2023). The EssenCES items were professionally translated and back-translated, with discrepancies resolved through consultation with the first author.

### Sample

Data were collected anonymously from 759 participants (576 staff, 183 inmates). After excluding 18 cases with incomplete responses on the EssenCES subscales, the final sample included 741 individuals: 598 from prisons (478 inmates, 120 staff) and 143 from forensic hospitals (88 patients, 55 staff). Participants were drawn from two maximum-security prisons (Arad *N* = 145; 31 staff; 114 inmates and Craiova N = 148; 30 staff, 118 inmates), two semi-open prisons (Timișoara *N* = 150; 30 staff; 120 inmates and Satu Mare *N* = 155; 29 staff; 126 inmates), and two forensic hospitals (București-Dej *N* = 85; 26 staff; 59 patients and Mioveni *N* = 58; 29 staff; 29 patients). The sample met the minimum recommended number of 7–10 questionnaires per ward (Schalast and Tonkin, 2016). All participants had been in custody or employed at their respective institutions for at least 2 months prior to data collection.

## Measures

### Prison social climate

EssenCES (Schalast and Tonkin, 2016) contains 17 items featuring the three subscales patients’/inmates’ cohesion PH, experienced safety (ES), and therapeutic hold (TH), each consisting of five items. Items 1 and 17 are unscored filler items. The 5-point answer scale ranges from 0 (not at all) to 4 (very much), producing sum scores ranging from 0 to 20, positive values being indicative of a positive social climate.

### Working environment

The Working Environment Scale (WES-10; Røssberg et al., 2004) is a 10-item instrument assessing perceptions of the work environment across four domains: self-realization, workload, conflict, and nervousness. Items are rated on 5-point Likert scales, with varying anchors. Lower scores reflect a positive work climate—low stress, high morale, and growth potential—whereas higher scores indicate elevated conflict, stress, and limited opportunities for development. In this study, WES-10 scores ranged from 1.00 to 3.90 (*M* = 2.40, SD = 0.60).

### Institutional aggression

Aggression was measured by the number of staff-recorded incidents over the 2 months prior to data collection, consistent with previous validation studies (Tonkin et al., 2012; Long et al., 2010).

### Procedure

Participants were invited to complete the questionnaires anonymously and confidentially within the prison or forensic hospital. Participation was voluntary, with informed consent obtained prior to completion. Completed forms were returned to the unit psychologist, who forwarded anonymized responses to the research team.

### Statistical analyses

Multivariate analysis of variance (MANOVA) was performed using IBM SPSS Statistics version 23. Other statistical analyses were performed in R version 4.4.1 (R Core Team, 2024).

To import the data, we used the *haven* package from R (Wickham et al., 2023). For data manipulation and transformation, we used the *tidyverse* collection of packages from R (Wickham et al., 2019). The analyses were run using prorated data. The prorating procedure (Schalast and Tonkin, 2016) is a method for dealing with missing data as follows: if four out of the five items were present for an individual on a single scale, the missing item was replaced with the mean for that individual. But if more than one item were missing per five-item scale, all the items for that scale were classed as missing for that participant. Notably, 64 cases (10.70%) out of 598 from prisons and six cases (4.20%) out of 143 from forensic hospitals required pro-rating. Overall, 70 cases (9.44%) out of 741 reported an item missing per at least one of the three 5-item scales.

Confirmatory factor analyses (CFAs) using maximum likelihood (ML) as the estimation method were employed using the *lavaan* package from R (Rosseel, 2012). CFA was used to examine the factorial structure of the Romanian version of the EssenCES. A one-factor solution and a three-factor solution model were tested. Model fit was evaluated using Chi-square statistics and alternative fit indices, including comparative fit index (CFI), Tucker-Lewis index (TLI), and root-mean-square error of approximation (RMSEA). We followed the literature recommendations in assessing models in terms of their goodness of fit (Bentler, 1990; Byrne, 1998; Hu and Bentler, 1999). Thus, adequate models should have a CFI and a TLI higher than 0.90, an RMSEA, and a standardized root-mean-squared residual (SRMR) below 0.08, but preferably lower than 0.05. Similarly, a ratio for *χ*^2^ to the degrees of freedom of less than 2.00 indicates a good fit, whereas a value less than 3.00 suggests an acceptable fit. For model comparison, Δ*χ*^2^ was used (if Δ*χ*^2^ is significant, the fit of the second model is significantly worse than the fit of the first model). However, since Δ*χ*^2^ is sensitive to sample size, alternative criteria such as the Akaike information criterion (AIC) and Bayesian information criterion (BIC) were used, with lower values indicating better model fit.

We conducted sequential invariance models (configural, metric, and scalar) using a stepwise method to test each measurement invariance (MI). *lavaan* and *semTools* (Jorgensen et al., 2022) packages from R were employed for this purpose. Multigroup CFA was conducted to investigate the MI of the scale across different types of settings (prisons vs. hospitals) and participants (residents vs. staff members). The configural model concerned whether the dimensionality and the pattern of factor-item relationships in the EssenCES were identical between the setting groups (prisons vs. hospitals) and types of participants (residents vs. staff members). While holding the general latent structure equal, the configural models allowed item loadings and thresholds to be freely estimated within each group. In the metric invariance model, loadings of each item were set to be equal across settings groups and participant groups, but item thresholds were freely estimated within each group. The scalar invariance models imposed additional constraints on the item thresholds (item intercepts). The equivalence of measurement models across different types of settings (prisons vs. hospitals) and participants (residents vs. staff members) was evaluated by the magnitude of changes in the referred model fit indices (*Δ*CFI ≤ −0.01, Cheung and Rensvold, 2002; Δ RMSEA <−0.015, Chen, 2007). A ΔCFI less than or equal to 0.01 indicates that the more constrained model does not significantly worsen the fit compared to the less constrained model. A ΔRMSEA less than or equal to 0.015 suggests that the more constrained model does not significantly degrade the fit compared to the less constrained model, indicating invariance.

*SemTools* and *psych* (Revelle, 2024) packages were used to calculate average variance extracted (AVE), internal consistency (Cronbach’s alpha), and other reliability coefficients (McDonald’s omega). Linear regression models were obtained using the *stats* package (built in R), and correlations were performed using the *psych* package. We computed the intraclass correlation coefficients ICC(1) and ICC(2) as well as the within-group agreement index rWG(J) (James et al., 1984) for the EssenCES total score and its three subscales (patients’ cohesion, experienced safety, and therapeutic hold). ICC(1) estimates the proportion of variance in individual scores attributable to group membership (i.e., between-group heterogeneity), whereas ICC(2) reflects the reliability of group means (Turhan et al., 2024; Van der Helm et al., 2024). The rWG(J) index assesses the agreement among respondents within the same group on multi-item scales. Values ≥ 0.70 generally indicate good within-group agreement (Bliese, 2000). Analyses were conducted separately for each security level (semi-open prison, maximum-security prison, and forensic hospital), with participant (inmate/patient vs. staff member) as the grouping variable. All computations were performed in R using the HLM_ICC_rWG(j) function from the *bruceR* package (Bao, 2023).

Multilevel modeling using linear mixed-effects models (LMMs) was performed using *lme4* package from R (Bates et al., 2015). A series of LMMs was fitted to examine whether scores on EssenCES subscales (therapeutic hold, experienced safety, and patients’ cohesion) vary as a function of institutional aggression, security level, occupational position (staff member or resident), and their interactions. Two sets of models were specified for each EssenCES subscale. The first estimated institutional aggression, occupational position (staff member vs. resident), and their interaction as fixed effects. In the second one, we replaced institutional aggression with security level, retaining occupational position and its interaction with security level as fixed effects. All models also included a random intercept for estate to account for the nesting of responses within institutions. We used the institution (estate) as the cluster variable to account for the variance in responses arising from participants being nested within the same facility. This approach statistically controls for shared environmental, procedural, and organizational factors within each institution that could systematically influence EssenCES subscale ratings, beyond individual-level predictors. Although all institutions operate under the same national regulations and policies, each facility develops its own operational procedures and organizational culture. These facility-specific characteristics and practices, such as staff–inmate interaction styles or the overall organizational climate, can exert a systematic influence on participants’ perceptions and experiences, including their ratings on the EssenCES subscales. Marginal and conditional *R^2^* values were reported to reflect the variance explained by fixed effects and the whole model. *lmerTest* package (Kuznetsova et al., 2017) was employed to compute *p*-values for mixed models, and the *sjPlot* package (Lüdecke, 2024) from R was employed to create tables of model results. *ggplot2* (Wickham, 2016) and *effects* (Fox and Weisberg, 2019) packages from R were used for data visualization.

## Results

Table 1 presents the results of the confirmatory factor analysis for the Romanian EssenCES.

**Table 1:** The goodness-of-fit statistics for the four tested models using ML as the method of parameter estimation.

Model	*χ* ^2^	Df	Δ*χ*^2^	Δdf	CFI	TLI	RMSEA (90% CI)	SRMR	AIC	BIC
M1	269.75	87	–	–	0.941	0.929	0.053 (0.046, 0.061)	0.063	31,553	31,706
M2	1239.43	90	969.68^***^	3	0.627	0.565	0.131 (0.125, 0.138)	0.120	32,517	32,655

M1 = three-factor model with SC = PC + ES + TH; M2 = one-factor model; SC = Social Climate; *χ*^2^ = Chi-square; df = degrees of freedom; CFI = comparative fit index; TLI = Tucker-Lewis index; RMSEA = root-mean-square error of approximation; and SRMR = standardized root-mean-squared residual; ***p < 0.001. *N* = 741.

The results show that the three-factor model, which refers to the original three-factor solution proposed by the EssenCES authors, is the best-fitting solution. In this model, the three factors of social climate (SC) are patients’ cohesion (PC), experienced safety (ES), and therapeutic hold (TH). This finding supports the theoretical factor structure of the EssenCES. The measurement model shows adequate fit [*χ*^2^(87) = 269.75, RMSEA = 0.053, SRMR = 0.063, CFI = 0.941, TLI = 0.929], all standardized factor loadings are statistically significant and most of them are greater than 0.40, ranging from 0.44 to 0.79. Only items 15 (“Some patients are so excitable that one deals very cautiously with them./Some inmates are so excitable that one treats them very cautiously.”) and 13 (“Often, staff seem not to care if patients succeed or fail in treatment./Staff often seem not to care if the inmates succeed or fail in their daily routine/schedule.”) loaded poorly on their corresponding factors (0.29 and 0.21). However, the estimates were statistically significant (*p* < 0.001). Hence, we decided to keep all 15 items to ensure the scale’s cross-cultural equivalence. The standardized factor loadings for the original three-factor model of the EssenCES, along with individual item loadings, are reported in Table 2.

**Table 2:** Standardized factor loadings of the original three-factor model of EssenCES and item descriptive statistics.

Item Number	Item content (hospitals)	Item content (prisons and correctional settings)	PC	ES	TH	M	SD
EssenCES02	The patients care for each other.	Inmates take care of each other.	0.67			1.90	1.08
EssenCES05	Even the weakest patient finds support from his/her fellow patients.	Even the weakest inmate finds support from his fellow inmates.	0.73			2.17	1.11
EssenCES08	Patients care about their fellow patients’ problems.	Inmates care about their fellow inmates’ issues.	0.69			1.87	1.09
EssenCES11	When patients have a genuine concern, they find support from their fellow patients.	When inmates have a sincere concern, they find support from their fellow inmates.	0.74			2.05	1.04
EssenCES14	There is good peer support among patients.	There is good mutual support among inmates.	0.77			1.90	1.01
EssenCES03	Really threatening situations can occur here.	Really threatening situations may occur here.		0.68		1.96	1.28
EssenCES06	There are some really aggressive patients on this ward.	There are really aggressive inmates in this unit.		0.78		1.99	1.30
EssenCES09	Some patients are afraid of other patients.	Some inmates are afraid of other inmates.		0.69		2.09	1.19
EssenCES12	At times, members of staff are afraid of some of the patients.	Sometimes staff feel threatened by some inmates.		0.54		2.92	1.13
EssenCES15	Some patients are so excitable that one deals very cautiously with them.	Some inmates are so excitable that one treats them very cautiously.		0.29		2.13	1.07
EssenCES04	On this ward patients can openly talk to staff about all their problems.	In this unit, inmates can talk openly with the staff about all their issues.			0.64	2.67	1.12
EssenCES07	Staff take a personal interest in the progress of patients.	Staff takes personal interest in the inmates’ evolution.			0.79	2.68	1.16
EssenCES10	Staff members take a lot of time to deal with patients.	Staff members book a lot of time to care for the inmates.			0.45	2.39	1.22
EssenCES13	Often, staff seem not to care if patients succeed or fail in treatment.	Staff often seem not to care if the inmates succeed or fail in their daily routine/schedule.			0.21	2.66	1.18
EssenCES16	Staff know patients and their personal histories very well.	Staff know the inmates and their personal history very well.			0.56	2.75	1.15

EssenCES, the Essen Climate Evaluation Schema; PC, patients’ cohesion; ES, experienced safety; TH, therapeutic hold; M, mean; SD, standard deviation. *N* = 741.

### Multigroup analysis of invariance

Results of the multigroup confirmatory factor analysis (CFA) investigating the MI of the Romanian EssenCES scale are summarized in Table 3.

**Table 3:** EssenCES measurement invariance.

Model	Penitentiaries (*n* = 598) versus forensic hospitals (*n* = 143)							
	*χ* ^2^	df	CFI	RMSEA (90% CI)	Δ*χ*^2^	Δdf	ΔCFI	ΔRMSEA
Configural	455.99	174	0.910	0.066 (0.059, 0.074)	–	–	–	–
Metric	479.11	186	0.907	0.065 (0.058, 0.072)	23.12^*^	12	0.003	0.001
Full scalar	537.35	198	0.892	0.068 (0.061, 0.075)	58.24^***^	12	0.015	0.003
Partial scalar	521.62	197	0.897	0.067 (0.060, 0.074)	42.51^***^	11	0.010	0.002

Model	Residents (*n* = 566) versus staff members (*n* = 175)							
	*χ* ^2^	df	CFI	RMSEA (90% CI)	Δ*χ*^2^	Δdf	ΔCFI	ΔRMSEA
Configural	394.44	174	0.930	0.058 (0.051, 0.066)	–	–	–	–
Metric	431.29	186	0.922	0.060 (0.052, 0.067)	36.85^**^	12	0.008	0.002
Full Scalar	537.99	198	0.892	0.068 (0.061, 0.075)	106.7^***^	12	0.030	0.008
Partial Scalar	470.52	194	0.912	0.062 (0.055, 0.069)	39.23^***^	8	0.010	0.002

***p < 0.001; **p < 0.01; *p < 0.05, *N* = 741.

In the first step, we tested configural invariance, which assumes equal factor structure among groups. Results of the MI analysis (Table 3) show that configural invariance was achieved for both group comparisons (prisons vs. hospitals and residents vs. staff members), meaning that the three-factor structure is equivalent across prison and hospital settings and across staff members and residents. In the next step, metric invariance was tested, so the factor loadings are constrained to be equal across groups. Results of the MI analysis (Table 3) show that the loadings are equivalent across groups, meaning that metric invariance was also confirmed. The items of the three-factor structure model have the same meanings in both groups (prisons vs. hospitals and residents vs. staff members). Scalar invariance requires equal item intercepts (or thresholds) across groups. Scalar invariance is not fully supported (see full scalar from Table 3). However, partial scalar invariance may still be possible and is often acceptable. We proceeded by identifying non-invariant items using modification indices. Consequently, we allowed Item 2 intercept to be free across the prisons and forensic hospitals groups. Finally, we freed the intercepts of Items 16 and 13 from therapeutic hold and Items 9 and 12 from experienced safety across the inmate and staff members groups. Results show partial scalar invariance was achieved for both group comparisons (Table 3), which justifies comparing latent means across groups.

## Intraclass correlations and the level of within-group rater agreement

The EssenCES scales measure group-level constructs across participant groups (e.g., staff and inmates/patients); therefore, intraclass correlations and within-group rater agreement rWG(j) are key for assessing score consistency within groups (Biemann et al., 2012). Consistent with previous prison climate research (Turhan et al., 2024; Van der Helm et al., 2024), we assessed intraclass correlations and interrater reliability for the EssenCES total scale and its three subscales—patient’s/inmate’s cohesion (PC), therapeutic hold (TH), and experienced safety (ES)—across two participant groups (staff and inmates/patients) and three security levels (semi-open access, maximum security, and forensic hospital). ICC(1)s were calculated for the EssenCES score and its three subscales (patients’ cohesion, experienced safety, and therapeutic hold) to examine the amount of variability in scores between groups, that is, to what extent variation in scores could be attributed to individuals versus the group level (inmates/patients vs. Staff members) within each security level. Smaller ICC(1) values indicate greater homogeneity within groups, while higher ICC(1) values suggest greater heterogeneity among groups. ICC(1) values ranged from 0.04 in maximum-security prisons to 0.28 in forensic hospitals, indicating that between 4 and 28% of the variance in scores could be attributed to the group level. For patients’ cohesion, ICC(1) values ranged from 0.03 in maximum-security prisons to 0.27 in forensic hospitals, for experienced safety from 0.045 in semi-open prisons to 0.476 in forensic hospitals, and for therapeutic hold from 0.186 in forensic hospitals to 0.358 in maximum-security prisons. The proportion of variance attributable to group membership was smallest for patients’ cohesion in maximum-security prisons (greater homogeneity between groups) and largest for experienced safety in secure hospitals (greater heterogeneity).

To examine the reliability of scores as a group construct, we computed ICC(2)s. The consistently high values (0.76–0.98) indicate good to excellent reliability of group means. rWG(J) values, also high, ranged from 0.72 to 0.93 and showed good to strong within-group agreement on items of the scales. The relatively high ICC(1) values further imply that a meaningful proportion of the variance in climate perceptions is attributable to group membership, justifying the aggregation of individual scores to the group level in subsequent analyses. Group sizes varied from 55 to 246 participants per subgroup, ensuring sufficient representation for each category. ICC(1), ICC(2), and rWG(J) values for all measures are presented in Table 4.

**Table 4:** Interclass correlations and interrater reliability within the group for EssenCES items.

EssenCES total score						
	Security level	Groups	*N*	ICC1	ICC2	rWG(J)
1	Semi-open prison	Staff members	59	0.126	0.934	0.928
		Inmates	246			
2	Maximum-security prison	Staff members	61	0.04	0.811	0.909
		Inmates	232			
3	Secure hospital	Staff members	55	0.281	0.964	0.915
		Patients	88			

PC (patients’ cohesion)						
	Security level	Groups	*N*	ICC1	ICC2	rWG(J)
1	Semi-open prison	Staff members	59	0.146	0.943	0.858
		Inmates	246			
2	Maximum-security prison	Staff members	61	0.029	0.76	0.797
		Inmates	232			
3	Secure hospital	Staff members	55	0.27	0.962	0.853
		Patients	88			

ES (experience safety)						
	Security level	Groups	*N*	ICC1	ICC2	rWG(J)
1	Semi-open prison	Staff members	59	0.045	0.829	0.763
		Inmates	246			
2	Maximum-security prison	Staff members	61	0.117	0.929	0.743
		Inmates	232			
3	Secure hospital	Staff members	55	0.476	0.984	0.725
		Patients	88			

TH (therapeutics hold)						
	Security level	Groups	*N*	ICC1	ICC2	rWG(J)
1	Semi-open prison	Staff members	59	0.322	0.979	0.802
		Inmates	246			
2	Maximum-security prison	Staff members	61	0.358	0.982	0.779
		Inmates	232			
3	Secure hospital	Staff members	55	0.186	0.94	0.754
		Patients	88			

ICC, intraclass correlation coefficient; rWG(J), within-group rater agreement for multi-item measures.

## Internal consistency

To examine the reliability of EssenCES scales, we calculated Cronbach’s alpha (*α*) and McDonald’s omega (*ω*) for each EssenCES factor. Given that the purpose of the scales is not to assess individual differences, but mean levels of groups of inmates or staff, the lowest observed internal consistencies of *α* = 0.55 can be considered as sufficient. For a satisfactory level of convergent validity, an average variance extracted value (AVE) greater than the threshold of 0.5 should be obtained for each scale (Fornell and Larcker, 1981). Although the AVE values for the experienced safety (ES) and therapeutic hold (TH) scales were less than 0.5 (Table 5), the fact that the scales demonstrated satisfactory internal consistency, alongside the fact that the measurement model fitted the data adequately. All standardized factor loadings are statistically significant, and the majority of them are greater than 0.40, confirming the construct validity (Cheung et al., 2023). To measure discriminant validity, we used the cross-loading technique (Fornell and Larcker, 1981). More specifically, discriminant validity is established when AVEs associated with two constructs are greater than the shared variance (SV; squared correlation) between the two constructs. All three scales—patients’ cohesion (PC), experienced safety (ES), and therapeutic hold (TH)—had average variance extracted values greater than the SV; therefore, the criterion for discriminant validity was met (Table 5).

**Table 5:** The correlation matrix for the three EssenCES scales, reliability coefficients, and descriptive statistics (mean, standard deviation, skewness, and kurtosis).

	Total sample (*n* = 741)									
	PC	ES	TH	*M*	SD	Skewness	Kurtosis	*α*	*ω*	AVE
PC	–			9.88	4.17	0.08	−0.26	0.84	0.84	0.51
ES	0.25^***^	–		11.09	4.18	−0.10	−0.48	0.74	0.76	0.41
TH	0.41^***^	0.045	–	13.15	3.75	−0.35	−0.13	0.64	67	0.31

	Prisons (*n* = 598)									
	PC	ES	TH	*M*	SD	Skewness	Kurtosis	*α*	*ω*	AVE
PC	–			9.54	4.13	0.08	−0.22	0.83	0.83	0.50
ES	0.20***	–		10.96	4.09	−0.10	−0.43	0.74	0.76	0.41
TH	0.47***	0.10*	–	12.78	3.73	−0.31	−0.14	0.65	0.68	0.33

	Forensic Hospitals (*n* = 143)									
	PC	ES	TH	*M*	SD	Skewness	Kurtosis	*α*	*ω*	AVE
PC	–			11.31	4.03	0.09	−0.48	0.85	0.85	0.53
ES	0.39***	–		11.64	4.53	−0.18	−0.66	0.76	0.77	0.43
TH	0.022	−0.26**	–	14.71	3.42	−0.53	0.21	0.55	0.60	0.25

	Residents (*n* = 566)									
	PC	ES	TH	*M*	SD	Skewness	Kurtosis	*α*	*ω*	AVE
PC	–			9.71	4.29	0.16	−0.38	0.83	0.83	0.49
ES	0.25***	–		11.67	4.09	−0.20	−0.30	0.71	0.73	0.38
TH	0.47***	0.20***	–	12.35	3.58	−0.33	−0.008	0.55	0.61	0.28

	Staff members (*n* = 175)									
	PC	ES	TH	*M*	SD	Skewness	Kurtosis	*α*	*ω*	AVE
PC	–			10.43	3.69	−0.21	0.50	0.89	0.89	0.62
ES	0.37***	–		9.22	3.92	0.19	−0.61	0.77	0.79	0.46
TH	0.19*	0.008	–	15.76	3.04	−0.59	−0.10	0.71	0.72	0.36

EssenCES, the Essen Climate Evaluation Schema; PC, patients’ cohesion; ES, experienced safety; TH, therapeutic hold; ***p < 0.001, **p < 0.01, *p < 0.05.

Overall, it can be concluded that all EssenCES scales have an appropriate degree of reliability and validity. The reliability estimates for the EssenCES’s scales were mostly acceptable, ω ranging from 0.60 to 0.89 (omega is more suitable than alpha for assessing reliability in complex measurement models, such as multidimensional scales, especially when the assumption of tau-equivalence is violated; Cheung et al., 2023). The values ranging from 0.60 to 0.72 were registered for the TH (Table 5) scale, and in the case of inmates/patients, values under 0.70 were recorded. As posited in other studies (Chester et al., 2015; Robinson and Craig, 2019), the intricacies of language, particularly in relation to therapy involving abstract concepts, double negatives, or complex language, are likely to be a contributing factor to the difficulties in comprehending the items. As was the case with the Australian sample, as demonstrated by Day et al. (2012), analogous aspects were encountered. The overall reliability score for EssenCES’s alpha, as rated by patients, was 0.64, whereas for the ES scale it was 0.62 (Day et al., 2012). Values of 0.60 and above may be used for short scales when aggregated across groups. The Romanian version of the EssenCES is a reliable and valid measurement of the social climate of correctional settings. Overall, the results show that PC has the highest AVE (0.51), explaining 51% of the variance, followed by ES (0.41) explaining 41% of the variance and finally by TH (0.31) explaining 31% of the variance. This structure is nearly identical for the whole sample (741 respondents) and for the subdivisions (prisons vs. hospitals and inmates vs. staff) (Table 5).

### Predictive validity

In order to test predictive validity, we used the WES-10, the number of aggressive incidents and site security, consistent with previous validation studies (Tonkin et al., 2012; Day et al., 2012). The relationship between scores on the three EssenCES scales and the scores on the WES-10 was examined for staff members (*n* = 175), with the three EssenCES scales predicting the total WES-10 score. The data distribution is normal, with a skewness of 0.10 [standard error (SE) = 0.18] and a kurtosis of −0.17 (SE = 0.37). The WES-10 had Cronbach’s *α* of 0.75, indicating a good internal consistency.

The results of the regression analysis showed that patients’ cohesion significantly predicted a negative working environment (*β* = −0.20, *p* = 0.004, *p* < 0.01), accounting for 3.4% of its variance (*R*^2^ = 0.034) when the other two predictors were controlled for. Experienced safety significantly predicted a negative working environment (*β* = −0.27, *p* < 0.001), explaining 6.5% of variance (*R*^2^ = 0.065) when the other two predictors were controlled. Therapeutic hold significantly and negatively predicted a negative working environment (*β* = −0.38, *p* < 0.001), explaining 13.7% of its variability (*R*^2^ = 0.137) when the other two predictors were controlled. All three predictors simultaneously accounted for 33.4% (*R*^2^ = 0.334) of the variance in the working environment. Staff members who perceived a more positive social climate tended to report working in an environment where morale was high and stress is low. Staff who rated their ward/wing as safe and therapeutically supportive with the EssenCES also tended to rate their working environment in a positive manner. The strongest predictor out of the three EssenCES scales was therapeutic hold.

### Institutional aggression

From the 742 participants’ inmates and staff, 581 (78,4%) reported the number of incidents for the last 2 months and 160 (21,6%) answers were missing. Out of the 78,4%, 72,9% (540 participants) reported between 0 and 2 incidents. Due to the high number of missing responses from staff members regarding institutional aggression (97 missing responses out of 175 participants), we explored the relationship between institutional aggression and the three dimensions of EssenCES only in the resident sample (inmates and patients; *n* = 503). Since the self-reported number of aggressive incidents recorded during the 2 months preceding data collection was highly skewed (skewness = 9.07), we used Spearman’s *ρ* correlation coefficient. The results indicate a negative and statistically significant correlation between the number of reported aggressive incidents and the three dimensions of EssenCES [PC and institutional aggression *ρ*(501) = −0.09, *p* < 0.05; ES *ρ*(501) = −0.16, *p* < 0.001; TH *ρ*(501) = −0.095, *p* = 0.02, *p* < 0.01]. A higher number of aggressive incidents was related to lower scores of patients’ cohesion, experienced safety, and therapeutic hold. Therefore, the results show that a more positive social climate in the prison is related to less aggressive incidents. On wards/wings where participants perceived a low level of aggression, they tended to feel more safe, more cohesive, and more supportive with each other.

## Multilevel analysis

### Institutional aggression

**Figure 1:**
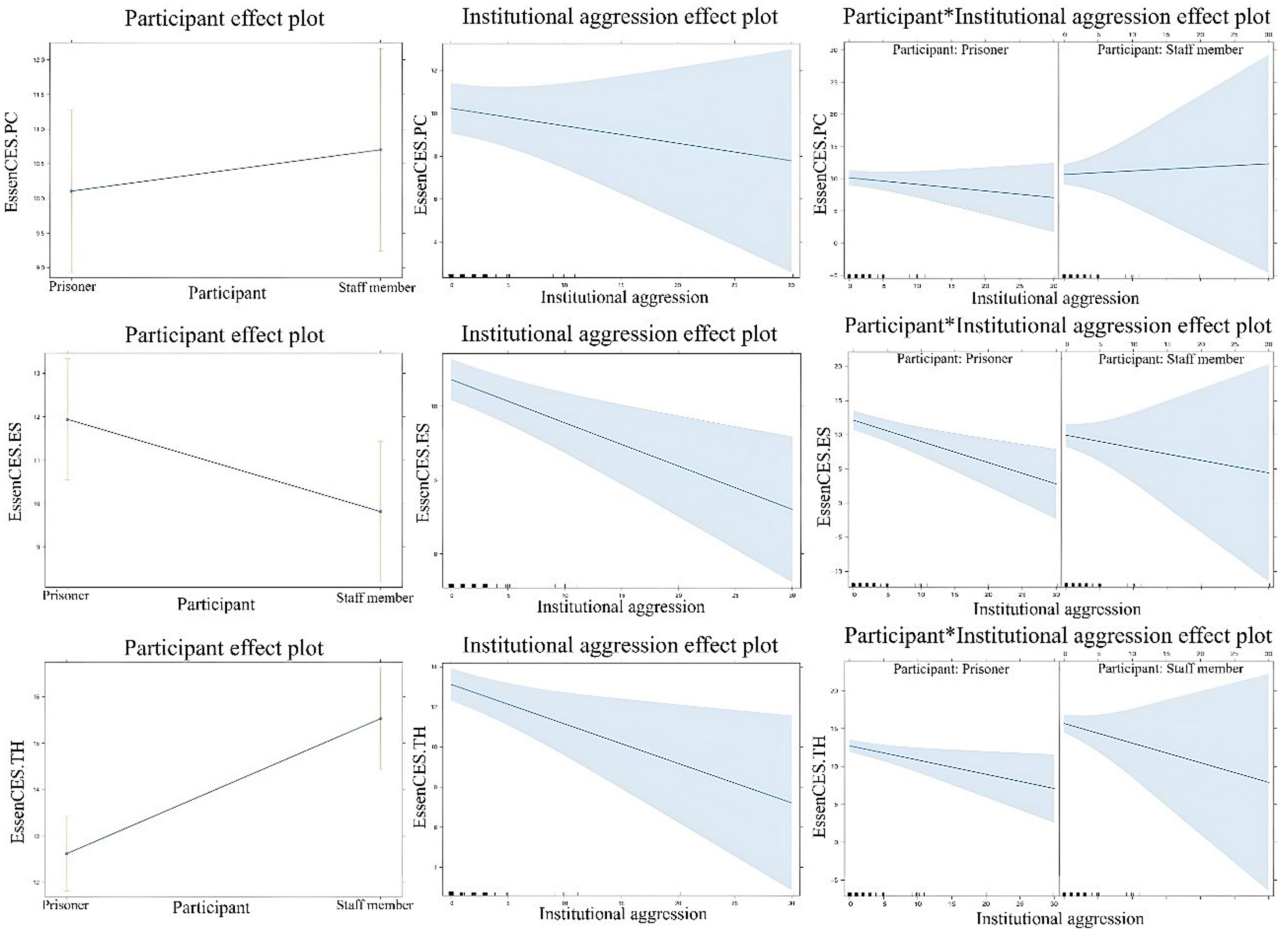
Institutional aggression and EssenCES scales multilevel analysis.

Mixed-effects models assessed whether each EssenCES scale score (Table 6) varied as a function of institutional aggression and whether occupational position (staff member or resident) influenced these ratings. Higher institutional aggression was significantly associated with lower experienced safety (ES) and therapeutic hold (TH) scores. Additionally, staff members rated safety 2.19 points lower than residents and therapeutic hold 2.94 points higher than residents. No significant interactions were found, meaning institutional aggression had a consistent effect across occupational positions. These findings suggest that institutional aggression negatively impacts perceptions of safety and therapeutic climate, independent of occupational position; both staff members and residents perceive this relation similarly. The images of the multilevel analysis of aggression and EssenCES scales can be seen in Figure 1.

**Table 6:** Institutional aggression and EssenCES scales mixed-effects model.

Predictors	EssenCES therapeutic hold						EssenCES experienced safety						EssenCES patients’ cohesion					
	Estimates	Std. error	CI	Statistic	*p*	df	Estimates	Std. error	CI	Statistic	*p*	df	Estimates	Std. error	CI	Statistic	*p*	df
(Intercept)	12.73	0.41	11.92–13.53	31	<0.001	575	12.11	0.71	10.72–13.51	17.08	<0.001	575	10.16	0.6	8.99–11.34	17.01	<0.001	575
Participant (staff member)	2.95	0.49	1.98–3.91	6	<0.001	575	−2.19	0.54	−3.26 – −1.12	−4.03	<0.001	575	0.51	0.58	−0.63 – 1.65	0.88	0.38	575
Institutional aggression	−0.19	0.08	−0.34 – −0.04	−2.45	0.015	575	−0.31	0.08	−0.48 – −0.14	−3.67	<0.001	575	−0.1	0.09	−0.28 – 0.08	−1.13	0.257	575
Participant (staff member) * Institutional aggression	−0.07	0.26	−0.58 – 0.44	−0.27	0.785	575	0.13	0.29	−0.44 – 0.70	0.44	0.662	575	0.16	0.31	−0.45 – 0.76	0.51	0.609	575
Random effects																		
σ^2^	12.06						14.76						16.84					
τ_00 Estate_	0.83						2.8						1.89					
ICC	0.06						0.16						0.1					
*N*_Estate_	6						6						6					
Observations	581						581						581					
Marginal *R*^2^/Conditional *R*^2^	0.076/0.136						0.050/0.201						0.004/0.105					

Estimates = fixed-effect coefficients; std. Error = standard error; CI = 95% confidence interval; Statistic = test statistic (t-value); p = p-value indicating statistical significance; df = degrees of freedom; σ^2^ = residual variance (within-group variance); τ₀₀ Estate = between-group (estate-level) variance; ICC = intraclass correlation coefficient (proportion of variance explained by grouping structure); N Estate = number of groups (estates); Observations = number of responses; Marginal *R*^2^ = proportion of variance explained by fixed effects only; Conditional R^2^ = proportion of variance explained by both fixed and random effects.

### Level of security

**Figure 2:**
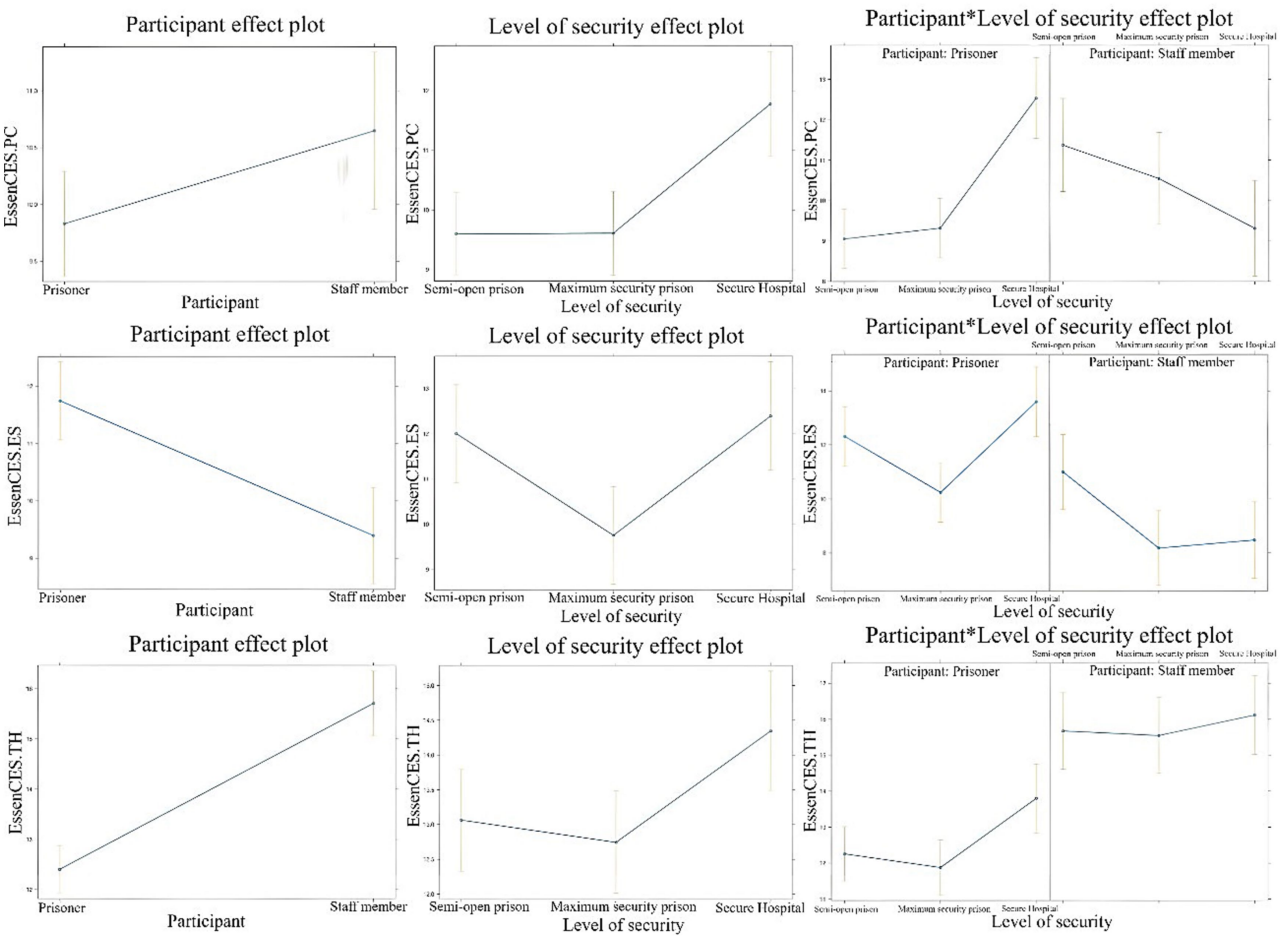
Level of security and EssenCES scales multilevel analysis.

Mixed-effects models assessed whether each EssenCES scale score (Table 7) varied as a function of occupational position and whether the level of security (semi-open prisons, maximum-security prisons, and forensic hospitals) influenced these ratings. Staff members rated safety (ES) 1.31 points lower than residents, therapeutic hold (TH) 3.42 points higher than residents, and cohesion (PC) 2.32 points higher than residents. Across groups, participants from forensic hospitals rated cohesion (PC) 3.48 points higher than participants from semi-open and maximum-security prisons. There is also a marginally significant fixed effect of the level of security on therapeutic hold (TH), with participants from forensic hospitals rating this dimension 1.54 points higher than participants from semi-open and maximum-security prisons. Another marginally significant fixed effect was obtained on experienced safety (ES), with participants from maximum-security prisons rating this dimension 2.07 points lower than their counterparts from semi-open and forensic hospitals. A significant interaction was found between occupational position and the level of security, with staff members from forensic hospitals rating cohesion (PC) and experienced safety (ES) 5.54, respectively, 3.82 points lower than inmates from forensic hospitals. The effect of occupational position on therapeutic hold was similar across the levels of security. The images of the multilevel analysis of site security and EssenCES scales can be seen in Figure 2.

**Table 7:** Level of security and EssenCES scales mixed-effects model.

Predictors	EssenCES patients’ cohesion						EssenCES experienced safety						EssenCES therapeutic hold					
	Estimates	Std. error	CI	Statistic	*p*	df	Estimates	Std. error	CI	Statistic	*p*	df	Estimates	Std. error	CI	Statistic	*p*	df
(Intercept)	9.05	0.37	8.32–9.78	24.37	<0.001	733	12.31	0.56	11.21–13.41	21.97	<0.001	733	12.26	0.38	11.50–13.01	31.86	<0.001	733
Participant (staff member)	2.32	0.58	1.18–3.46	4.01	<0.001	733	−1.31	0.55	−2.40 – −0.23	−2.37	0.018	733	3.42	0.49	2.45–4.39	6.93	<0.001	733
Level of security: maximum security	0.27	0.53	−0.77 – 1.30	0.5	0.615	733	−2.07	0.79	−3.63 – −0.51	−2.61	0.009	733	−0.38	0.55	−1.45 – 0.69	−0.69	0.488	733
Level of security: forensic hospital	3.48	0.63	2.25–4.72	5.52	<0.001	733	1.29	0.86	−0.40 – 2.98	1.5	0.135	733	1.54	0.62	0.32–2.76	2.49	0.013	733
Participant (staff member) * Level of security (maximum security)	−1.09	0.82	−2.70 – 0.51	−1.34	0.181	733	−0.75	0.78	−2.28 – 0.78	−0.96	0.337	733	0.25	0.69	−1.11 – 1.62	0.36	0.717	733
Participant (staff member) * Level of security (forensic hospital)	−5.54	0.9	−7.32 – −3.77	−6.14	<0.001	733	−3.82	0.87	−5.52 – −2.11	−4.4	<0.001	733	−1.1	0.77	−2.61 – 0.41	−1.43	0.152	733
Random effects																		
σ^2^	15.98						14.63						11.57					
τ_00 Estate_	0.15						0.51						0.2					
ICC	0.01						0.03						0.02					
*N*_Estate_	6						6						6					
Observations	741						741						741					
Marginal *R*^2^/Conditional *R*^2^	0.080/0.088						0.151/0.179						0.173/0.187					

Estimates = fixed-effect coefficients; std. Error = standard error; CI = 95% confidence interval; Statistic = test statistic (*t*-value); p = *p*-value indicating statistical significance; df = degrees of freedom; σ^2^ = residual variance (within-group variance); τ₀₀ Estate = between-group (estate-level) variance; ICC = intraclass correlation coefficient (proportion of variance explained by grouping structure); N Estate = number of groups (estates); Observations = number of responses; Marginal *R*^2^ = proportion of variance explained by fixed effects only; Conditional *R*^2^ = proportion of variance explained by both fixed and random effects.

**Figure 3:**
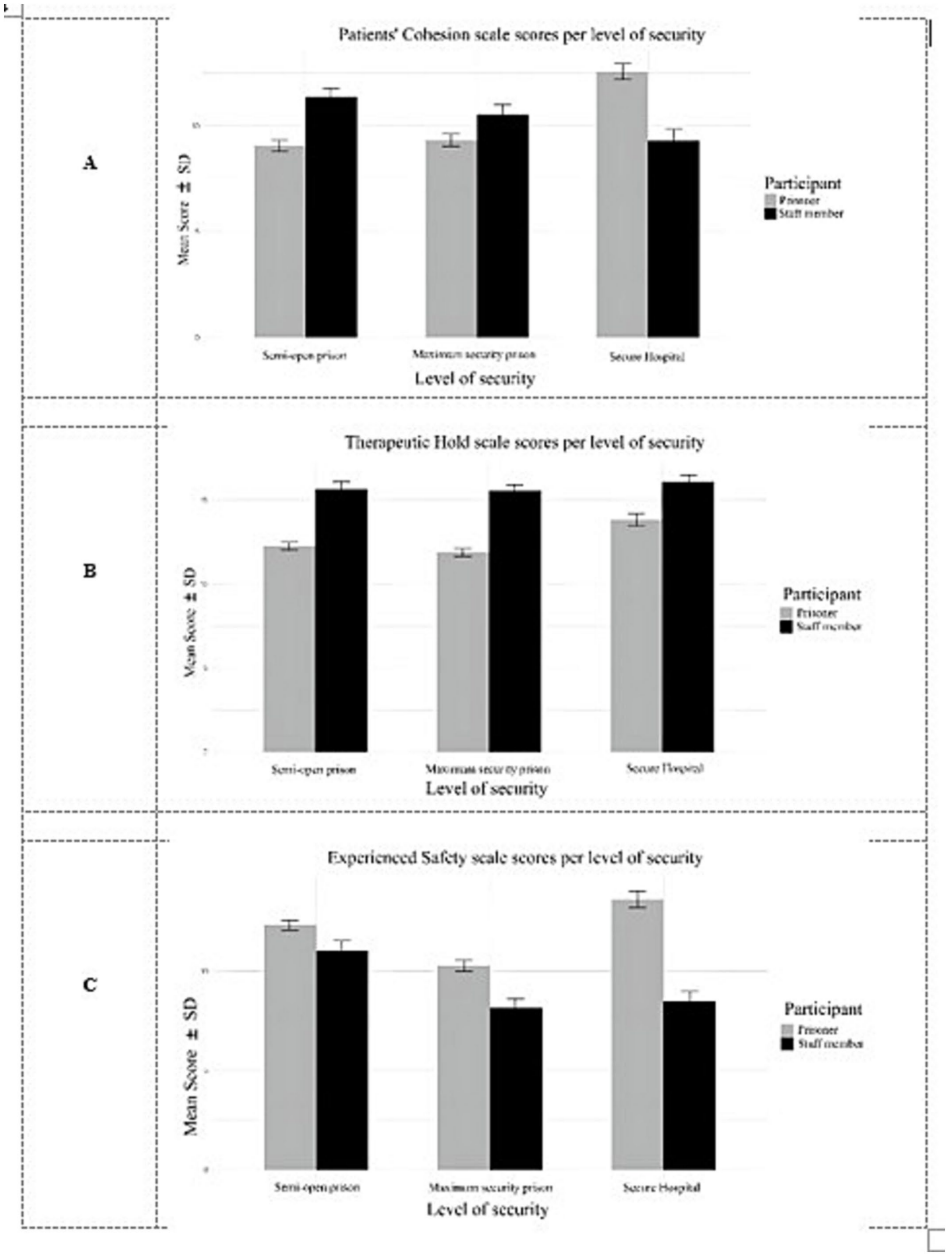
Variance of results for patients cohesion **(A)**, therapeutic hold **(B)** and experienced safety **(C)** scales across forensic facility.

A one-way MANOVA was conducted to assess the impact of security level (semi-open prison, maximum-security prison and forensic hospital) and one of the two groups (staff and inmates) on the prison climate (patients’ cohesion, experienced safety, and therapeutic hold). As Levene’s test for PC was significant (*p* = 0.009), the homogeneity criteria were not met for inmate’s/patients’ cohesion, hence it does not change the main results of the analysis, so we decided not to exclude the scale from the analysis of variance (see also Figure 3A). Therefore, the dependent variables were patients’ cohesion (PC) (Figure 3A), therapeutic hold (TH) (Figure 3B), and experienced safety (ES) (Figure 3C), EssenCES scale scores.

We examined *a priori* the assumptions of multivariate outliers, multivariate normality, homogeneity of variance–covariance matrices, and absence of multicollinearity. A Mahalanobis distance analysis was conducted to detect multivariate outliers. One case exceeded the critical *χ*^2^ threshold [*χ*^2^(3) = 16.27, *p* < 0.001] with a distance of 17.427, suggesting a potential multivariate outlier. Sensitivity analyses indicated that this case did not substantially alter the results, so it was retained in further analysis. Box’s test is not significant (*p* = 0.055), and also the Levene test for ES (*p* = 0.88) and TH (*p* = 0.36) were above the threshold of 0.05, so the assumption of homogeneity is met. This indicates that both security levels and participant type have a significant effect on the combined dependent variables of the experienced safety (ES) and therapeutic hold (TH) scale scores.

Analyses were conducted using two dependent variables (ES and TH) and, in a separate model, three dependent variables (ES, TH, and PC) against violations. It was hypothesized that respondents across categories would demonstrate distinct yet converging perspectives, reflecting a shared understanding of the items, across the three EssenCES scales within all facility types. The results indicated generally convergent views, although ES and TH scores differed across security levels and between staff and inmates/patients within the same facility type. PC was not significant on its own but reached significance when both security level and participant category were considered simultaneously.

Multivariate analyses (Table 8) indicated significant differences in prison climate perceptions across levels of security, Wilks’ *Λ* = 0.93, *F*(6, 1,466) = 8.62, *p* < 0.001, partial η^2^ = 0.03, suggesting that perceptions varied by institutional security level. A significant multivariate effect of participant category (staff vs. inmates/patients) was also observed, Wilks’ *Λ* = 0.78, *F*(3, 733) = 67.82, *p* < 0.001, partial *η*^2^ = 0.21, indicating a large effect of participant role on overall climate perceptions. Furthermore, the interaction between participant category and security level was significant, Wilks’ *Λ* = 0.93, *F*(6, 1,466) = 5.62, *p* < 0.001, demonstrating that the relationship between participant role and climate perceptions differed depending on the level of security. Follow-up univariate analyses of the between-subjects effects revealed several significant findings. There was a significant main effect of security level on: experienced safety, *F*(2, 735) = 20.53, *p* < 0.001, partial *η*^2^ = 0.05, and therapeutic hold, *F*(2, 735) = 5.80, *p* = 0.003, partial *η*^2^ = 0.01. However, security level did not significantly affect patients’ cohesion (*p* > 0.05).

**Table 8:** Multivariate testsª MANOVA results.

Effect	Test statistic	Value	*F*	df	Error df	Sig.	Partial *η*^2^	Non-centrality parameter
Intercept	Wilks’ Lambda	0.06	3790.59	3	733	< 0.000	0.939	11371.78
Level of security	Wilks’ Lambda	0.93	8.62	6	1,466	< 0.000	0.034	51.75
Participant	Wilks’ Lambda	0.78	67.82	3	733	< 0.000	0.217	203.47
Level of security*Participant	Wilks’ Lambda	0.93	5.62	6	1,466	< 0.000	0.034	51.23

*N* = 741. ^a^The values reported in this table are exact statistics.

A significant main effect of participant category (staff vs. inmate-patient) was also observed on: experienced safety, *F*(1, 735) = 68.65, *p* < 0.001, partial *η*^2^ = 0.08, and therapeutic hold, *F*(1, 735) = 105.08, *p* < 0.001, partial *η*^2^ = 0.12. These findings indicate that staff and inmate-patients differed in their perceptions of safety and therapeutic support. The interaction between security level and participant category was significant for patients’ cohesion, *F*(2, 735) = 20.56, *p* < 0.001, partial *η*^2^ = 0.06, and experienced safety, *F*(2, 735) = 10.38, *p* < 0.001, partial *η*^2^ = 0.02, but not for therapeutic hold (*p* = 0.16). These interaction effects suggest that the relationship between participant role and perceptions of the prison climate—particularly regarding cohesion and safety—varied depending on the security level of the institution.

We hypothesized that forensic hospitals would demonstrate higher perceived levels of TH and ES than high-security and semi-open wards. Conversely, we anticipated higher levels of PC in semi-open prisons and forensic hospitals than in high-security regimes. For this hypothesis, we tested individual mean differences across the three levels of security (semi-open prison, maximum-security prison, and forensic hospital) and EssenCES scales (experienced safety and therapeutic hold). The setting rated as safest was the semi-open one, followed by the forensic hospital and the maximum-security prison. On average, there were statistically significant differences in experienced safety perception both between maximum-security prison and semi-open prison (*p* < 0.001), and maximum-security prison and forensic hospital (*p* < 0.001), but not between forensic hospital and semi-open prison (*p* = 0.28). The results revealed that, on average, forensic hospital respondents reported significantly higher scores on the therapeutic hold scales than semi-open and maximum-security prison respondents, who had a similar appreciation of the therapeutic impact of the hold. The differences in the way participants rated the therapeutic environment of the hold were significant both between the forensic hospital and semi-open prison (*p* < 0.001) and hospital and maximum-security prison (*p* < 0.001). There were no statistically significant differences in therapeutic hold scores between maximum-security and semi-open prison respondents (*p* = 0.33).

In Table 9, we present preliminary normative data for the Romanian sample based on mean scores and standard deviations of the entire sample. Furthermore, the EssenCES climate ratings on the three scales in Romanian prisons demonstrated a closer alignment with those in England (Tonkin et al., 2012) and Germany (Schalast and Groenewald, 2009; Howells et al., 2009) than with those in Australia (Day et al., 2012).

**Table 9:** Comparison of sample characteristics, EssenCES scales.

		Romanian sample (present study)		German sample (Schalast and Groenewald, 2009)		The United Kingdom sample (Tonkin et al., 2012)		Australia sample (Tonkin et al., 2012)	
Sample	EssenCES Scales	Mean (SD)	*N*	Mean (SD)	*N*	Mean (SD)	*N*	Mean (SD)	*N*
Prison Inmates	PC	9.7 (4.2)	566	10.5 (3.9)	1175	11.5 (5.0)	139	13.4 (4.4)	123
	ES	11.6 (4.0)		13.3 (4.1)		13.3 (3.8)		16.4 (3.8)	
	TH	12.3 (3.5)		11.3 (4.8)		12.0 (4.6)		12.7 (4.2)	
Prison Staff	PC	10.4 (3.6)	175	10.2 (3.0)	82	10.9 (3.9)	125	12.8 (3)	109
	ES	9.2 (3.9)		12.6 (3.5)		10.9 (3.7)		15 (3.7)	
	TH	15.7 (3.0)		13.7 (3.8)		15.2 (3.1)		16.6 (3.3)	

PC, patients’ cohesion; ES, experienced safety; TH, therapeutic hold.

Furthermore, the mean values and standard deviations for each participant group (staff members and inmate/patient subjects) across the three scales (PC, ES, and TH), along with the corresponding security settings, can be found in Table 10.

**Table 10:** Means and standard deviations on EssenCES for the three types of facilities.

EssenCES scales	High-security prison		Semi-open prison		Forensic hospital	
	Staff (*N* = 61)	Inmate (*N* = 232)	Staff (*N* = 59)	Inmate (*N* = 246)	Staff (*N* = 55)	Patient (*N* = 88)
PC	10.5 (3.7)	9.3 (4.4)	11.3 (3)	9 (4)	9.3 (3.9)	12,5 (3.5)
ES	8.1 (3.5)	10.2 (3.9)	12.3 (3.8)	11 (4.03)	8.4 (3.6)	13.6 (3.8)
TH	15.5 (2.9)	11.8 (3.5)	15.6 (3)	12.2 (3.5)	16.1 (2.9)	13.8 (3.4)

PC, patients’ cohesion; ES, experienced safety; TH, therapeutic hold.

## Discussion

The establishment of (partial) scalar invariance of the EssenCES scales across varying levels of institutional security (i.e., high-security prisons, semi-open prisons, and forensic psychiatric hospitals) and participant roles (staff vs. inmate-patients) provides strong psychometric support for the measurement equivalence of the Prison Climate Scale across key forensic subgroups. Although full scalar invariance was not achieved, the attainment of partial scalar invariance nonetheless permits meaningful and unbiased comparisons of latent mean differences, thereby enhancing the construct validity of the instrument and supporting its applicability and comparability across diverse correctional and forensic contexts. This conclusion is further substantiated by the presence of strong configural invariance—indicating that the same underlying factor structure holds across all groups, with items consistently loading on their intended latent constructs—and by metric invariance, which demonstrates that the strength of the relationship between items and their corresponding factors is equivalent across groups.

From a forensic psychological perspective, these results have significant implications for both clinical practice and institutional policy. The ability to reliably assess the prison climate across heterogeneous populations facilitates the identification of environmental risk factors, supports routine institutional monitoring, and informs targeted interventions aimed at enhancing therapeutic engagement and perceived safety. For example, the lower ratings of safety reported by individuals in higher-security units may indicate the need for procedural or environmental modifications to improve psychological well-being and reduce perceived threat.

The confirmation of scalar invariance also strengthens the foundation for research examining the impact of institutional climate on mental health outcomes, staff burnout, and treatment responsiveness. It provides confidence in the comparability of findings between staff and inmate-patient populations—an essential consideration in forensic settings characterized by asymmetrical power dynamics and differing institutional roles. Future research should aim to replicate these findings in broader forensic populations, including juvenile detainees, forensic psychiatric outpatients, and in longitudinal studies, to evaluate the temporal stability of measurement properties and the consistency of climate perceptions following institutional change or reform efforts. Where full scalar invariance is not achieved, partial invariance models may still allow for valid interpretation, provided adequate item-level equivalence is maintained.

The internal consistency of the EssenCES scales and its three-factor structure with the factors patients’ cohesion, experienced safety and therapeutic hold developed by Schalast and Tonkin (2016) was confirmed in accordance with validation studies in Germany (Schalast et al., 2008), the United Kingdom (Tonkin et al., 2012; Milsom et al., 2014; Tomlin and Tonkin, 2023), and Australia (Day et al., 2012). For the lower internal consistency values of TH, as we argued before, values of 0.60 or higher may be considered acceptable for short scales, particularly when data are aggregated across groups. Two items (13 and 15) exhibited lower factor loadings; however, as these items belonged to distinct scales—therapeutic hold and experienced safety—their impact on the overall scale values was minimal. The words replaced with synonyms pertain to two fundamental psychological domains: motivation (e.g., success/failure for Item 13) and emotion (e.g., excitability for Item 15). Previous research (e.g., Tomlin and Tonkin, 2023) has suggested the revision of Items 10, 13, and 16 when applying the prison climate measure in security hospitals. In the current Romanian sample, the primary challenges were related to the comprehension of two concepts that were changed now to enhance item clarity and relevance in Romanian and similar contexts.

Given that the EssenCES evaluates aggregated group-level scores, we examined the proportion of variance in responses attributable to group membership (staff vs. inmates/patients) and the degree of interrater agreement. Within security levels, findings indicated that, for overall prison climate, a small proportion of variance was attributable to group membership in maximum-security settings, with higher proportions observed in semi-open prisons and forensic hospitals. This pattern was also observed for the prisoner–patient’ cohesion (PC) subscale. However, the experienced safety (ES) and therapeutic hold (TH) subscales displayed a different structure. Participants in the semi-open facility exhibited greater agreement regarding perceived safety, whereas responses from the forensic hospital were the most heterogeneous. These differences may be partially attributable to variation in residency duration: although all participants had spent at least 2 months in the facility, inmates generally resided longer in semi-open prisons than in forensic hospitals, where placements were more temporary. Overall, the TH subscale showed the greatest variability. Variability was lowest in the forensic hospital, with semi-open and high-security prisons displaying intermediate levels. This variability likely reflects differences in individual therapeutic needs across settings.

In general, the EssenCES instrument has been found to be a reliable tool for the evaluation of prison climate in both mainstream prisons and hospital units (Schalast and Groenewald, 2009; Tonkin et al., 2012; Day et al., 2012). It has demonstrated a high degree of adaptability to different cultural contexts (Tonkin, 2016; Tonkin et al., 2012; Day et al., 2012) and has been found to be an appropriate tool for the evaluation of the prison climate in Romania. The initial structure of the EssenCES was confirmed in the analysis of the Romanian prison and forensic hospital sample. As the findings indicate, both Romanian staff and inmate-patients reported generally positive perceptions of therapeutic support, suggesting a shared recognition of the availability and quality of psychological care within secure settings. These results are broadly consistent with findings from comparative studies conducted in the United Kingdom, reinforcing the cross-national validity of therapeutic climate assessments in forensic institutions and supporting the international relevance of constructs, such as therapeutic hold.

With regard to perceived cohesion, Romanian staff members tended to rate inmate-patient relationships as more cohesive than did the patients themselves. This pattern, consistent with previous international research, may reflect a role-related perceptual bias, whereby staff—observing from a position of authority—may view interpersonal dynamics through a more positive or optimistic lens than individuals embedded within those dynamics. Such differences underscore the importance of incorporating both staff and patient perspectives when evaluating institutional climate. Romanian staff participants reported lower levels of perceived safety than inmates/patients, consistent with patterns observed in prior studies from Germany, the United Kingdom, and Australia, where staff typically report similar or lower perceptions of safety compared with incarcerated individuals. Together, these findings highlight the need to interpret prison climate data within both cultural and occupational frameworks, and to consider how staff and patient roles shape perception. Cross-national comparisons also underscore the value of standardized climate assessments for guiding institutional reform and enhancing therapeutic outcomes across diverse correctional contexts.

In evaluating the construct validity of the three EssenCES subscales in relation to perceived working environment, site security, and institutional aggression, statistically significant associations were identified across all domains. Staff members who perceived a more positive social climate reported higher workplace morale and lower occupational stress. Specifically, wards perceived as both safe and therapeutically supportive were also described by staff as more favorable working environments. Of the three subscales, therapeutic hold emerged as the strongest predictor of perceived workplace quality. These findings align with previous research by Tonkin et al. (2012) and further support the role of therapeutic hold as a protective factor across both forensic psychiatric wards and correctional settings (Reading and Ross, 2020). These findings reinforce the view that a positive social climate—particularly one characterized by strong therapeutic relationships—plays a critical role in staff morale and psychological resilience. Enhancing elements of therapeutic hold may serve not only to benefit patients but also to reduce staff burnout and improve team cohesion in high-stress forensic environments.

Security level influenced how both staff and inmates perceived the social climate. Respondents from forensic psychiatric hospitals rated the environment as more therapeutic and cohesive than those from traditional prison settings. This aligns with prior research (e.g., Schalast and Laan, 2017; Efkemann et al., 2019), indicating that treatment-oriented units are perceived as more supportive by both staff and inmates. Therapeutic hold received the highest ratings from inmates in forensic psychiatric settings. Consistent with existing literature (Robinson and Craig, 2019), lower-security wards reported more favorable climate perceptions than high-security units.

Consistent with prior findings (Tonkin et al., 2012; Long et al., 2010), perceived ward aggression was negatively associated with both experienced safety (ES) and therapeutic hold (TH). Across roles, inmates and patients rated the environment as safer than staff—an effect observed internationally (e.g., Tonkin et al., 2012; Schalast and Tonkin, 2016). Patients perceived greater safety than inmates, and individuals in maximum-security settings—both staff and inmates—reported significantly lower safety perceptions than those in semi-open units. Elevated aggression was associated with a decline in perceived safety and therapeutic engagement, regardless of occupational role, highlighting the broader institutional impact of aggression on the prison climate. Differences in safety ratings by security level highlight the importance of environment-specific strategies to maintain therapeutic integrity in high-security units.

Establishing within-group agreement was important because it confirmed that climate perceptions represented shared views rather than isolated individual differences. Although staff and inmates/patients expressed perspectives that were distinct, these views were nonetheless aligned and convergent across settings, suggesting a common understanding of the institutional environment. In practice, this convergence highlights that interventions to improve prison climate may resonate across groups, even if their lived experiences differ. At the same time, the nuanced differences observed between regimes underscore the need for context-specific strategies, as therapeutic expectations in forensic hospitals may not fully align with safety concerns in high-security prisons or the community-oriented focus of semi-open facilities. Recognizing both the shared and distinct dimensions of climate perceptions can inform staff training, therapeutic programming, and institutional policy to enhance safety, cohesion, and rehabilitation outcomes across diverse correctional and forensic contexts.

Considering both shared and distinct climate perceptions, the three EssenCES scales collectively capture the multifaceted nature of prison climate. Although patients’ cohesion (PC) contributes to the overall perception of prison climate, findings in this study were inconsistent across settings. Prior research links PC to prosocial change and perceived safety (Williams et al., 2019; Dickens et al., 2014). In our sample, therapeutic hold (TH) and experienced safety (ES) showed consistent patterns: staff reported higher TH and lower ES than inmates across all security levels. However, PC varied by setting. Staff in semi-open and maximum-security prisons rated cohesion higher than inmates, while in forensic hospitals, staff rated it lower than patient-inmates. These discrepancies, supported by interaction effects in multilevel models and MANOVA results, may explain the violation of homogeneity assumptions for the PC scale. Divergent PC ratings suggest that staff-patient dynamics and perceived group cohesion vary significantly by institution type, with implications for team-based treatment approaches. These findings align with research highlighting the role of custodial therapeutic communities as structured, relationally focused interventions capable of addressing both criminogenic needs and psychological wellbeing (Richardson and Zini, 2021). Such settings have been associated with reduced recidivism and improvements in self-esteem, emotional regulation, and prosocial attitudes. When accompanied by a positive therapeutic climate, interventions targeting antisocial behavior have also demonstrated promising effects in lowering reoffending rates (Eltink et al., 2024).

Given prior associations between low cohesion and perceived risk, improving PC—particularly in forensic hospitals—may support both safer and more therapeutic environments.

A key finding was the divergence in ratings of experienced safety and therapeutic hold between staff and inmate-patients and across different security levels. Consistent with prior research (Williams et al., 2019), staff and residents perceived the prison climate differently. Respondents from forensic hospitals reported higher scores on both scales than those from semi-open and high-security prisons. This aligns with evidence that forensic settings are typically rated more positively than mainstream prisons (Reading and Ross, 2020). However, in line with Howells et al. (2009), higher scores in our study were observed only for therapeutic hold and experienced safety, not for all scales. As in previous studies (Siess and Schalast, 2017; Efkemann et al., 2019), therapeutic hold emerged as the most positively rated aspect of the prison climate among psychiatric forensic units. Moreover, consistent with earlier work (Milsom et al., 2014; Long et al., 2010), lower-security settings were associated with fewer incidents of aggression and greater therapeutic engagement. Lower-security institutions appear more conducive to therapeutic engagement and lower aggression, supporting efforts to adapt treatment frameworks to high-security environments.

### Limitations

The cross-sectional design employed in this study precludes drawing conclusions about causal relationships. Consequently, this study serves as the initial phase of a longitudinal investigation examining the effects of prison climate and risk factors on rehabilitation and recidivism. Future research will incorporate longitudinal structural equation modeling to explore causal inferences, including moderation and mediation effects. A limitation of this study is that we were only able to capture the perspectives of inmates/patients regarding aggressive incidents due to a high number of missing responses from staff members. Additionally, the study’s lack of female participants (both inmates and patients) limits the generalizability of findings to this subgroup. Finally, several considerations should be mentioned concerning the difference between the multigroup CFA sample sizes. When group sizes are unequal, the smaller group dictates the statistical power. Although the forensic hospitals (*n* = 143) and staff members (*n* = 175) subgroups were smaller than the penitentiary (*n* = 598) and inmates (*n* = 566) groups, prior simulation research indicates that multigroup CFA can yield robust, accurate results with subgroup sizes of approximately 100–150 when certain model characteristics are met (Meade and Bauer, 2007; Wolf et al., 2013). The adequacy of the sample size depends on the model complexity (e.g., the number of parameters to be estimated), communalities (e.g., higher than 0.50), factor loadings (e.g., higher than 0.70), number of groups (in this study, each specified model is divided into two groups), number of factors (three factors), items per factor (five items per factor), or missing-data handling (Meade and Bauer, 2007; Wolf et al., 2013). In the present study, our models involve a limited number of latent factors and a small, balanced set of items per factor. Standardized factor loadings were consistently strong, resulting in good communalities, which are known to enhance parameter stability even with smaller samples. Furthermore, the missing data were handled through prorating within the EssenCES scoring protocol, ensuring that no data points were lost in the estimation process. Together, these features are conditions under which methodological simulation studies have shown stable, acceptable fit indices, unbiased, accurate parameter estimates, and adequate power for invariance testing with subgroup sizes similar to those in the current study.

## Conclusion

The Romanian version of the EssenCES has demonstrated its effectiveness as a reliable tool for assessing prison climate across both mainstream prisons and forensic hospitals. Given its robustness, the EssenCES should be applied periodically to track changes in prison climate over time, helping institutions adapt their environments to the evolving needs of inmates.

Although individual ratings exhibited some variability—particularly among forensic hospital participants—aggregated scores demonstrated stable and reliable assessments of prison climate. Intraclass correlations indicated that a meaningful proportion of variance was attributable to group membership, supporting aggregation, while within-group agreement indices confirmed that raters within each group provided consistent evaluations. Overall, reliability was good to excellent across all three security levels, demonstrating that the EssenCES effectively captures both group-level perceptions and shared experiences within different custodial contexts.

Differences in perceptions of prison climate were observed between staff and inmates/patients, with staff reporting more positive views of therapeutic support, while inmates perceived safety more positively. This aligns with findings from previous studies. Notably, staff in semi-open and maximum-security prisons rated patients’ cohesion (PC) higher than their inmate counterparts, while staff in forensic hospitals rated PC lower than inmates in the same settings, as reflected in the interaction terms from the multilevel analyses.

The security level of the ward was found to significantly influence perceptions of the prison climate, with lower-security settings (i.e., semi-open and forensic hospitals) being viewed more positively, particularly in terms of therapeutic support. Maximum-security settings were associated with more negative perceptions of the environment. Differences in perceptions based on security levels suggest that interventions aimed at improving prison climate should be security-context specific, with an emphasis on enhancing therapeutic engagement in high-security settings.

Given the importance of prison climate in fostering inmate engagement in rehabilitation, the Romanian version of the EssenCES, with its three key scales (patients’ cohesion, experienced safety, and therapeutic hold), proves to be a valid and appropriate instrument for assessing prison climate in both mainstream prisons and forensic hospitals. A psychometrically sound evaluation of the prison climate can provide valuable insights for prison management and staff, enabling them to offer tailored support and create environments conducive to personal growth, therapeutic engagement, and the adoption of prosocial values.

## Data Availability

The data for statistical analysis is available upon request.

## References

[ref1] Bao H. (2023). bruceR: Broadly useful convenient and efficient R functions. R package version 2024.6. Available online at: https://cran.r-project.org/package=bruceR (Accessed August 15, 2025).

[ref2] Bates D. Maechler M. Bolker B. Walker S. (2015). Fitting linear mixed-effects models using lme4. J. Stat. Softw. 67, 1–48. doi: 10.18637/jss.v067.i01

[ref9012] Beech A. Fordham A. S. (1997). Therapeutic climate of sexual offender treatment programs. Sexual Abuse: A Journal of Research and Treatment 9, 219–237. doi: 10.1007/BF02675066 15974420

[ref3] Bennett J. Shuker R. (2018). Hope, harmony and humanity: creating a positive social climate in a democratic therapeutic community prison and the implications for penal practice [Hope, harmony and humanity]. J. Crim. Psychol. 8, 44–57. doi: 10.1108/JCP-06-2017-0030

[ref4] Bentler P. M. (1990). Comparative fit indexes in structural models. Psychol. Bull. 107, 238–246. doi: 10.1037/0033-2909.107.2.238, PMID: 2320703

[ref5] Biemann T. Cole M. S. Voelpel S. (2012). Within-group agreement: on the use (and misuse) of rWG and rWG (J) in leadership research and some best practice guidelines. Leadersh. Q. 23, 66–80. doi: 10.1016/j.leaqua.2011.11.006

[ref6] Bliese P. D. (2000). “Within-group agreement, non-independence, and reliability: implications for data aggregation and analysis” in Multilevel theory, research, and methods in organizations: foundations, extensions, and new directions. eds. Klein K. J. Kozlowski S. W. J. (San Francisco, CA: Jossey-Bass), 349–381.

[ref7] Bosma A. Q. van Ginneken E. Palmen H. Pasma A. J. Beijersbergen K. A. Nieuwbeerta P. (2020). A new instrument to measure prison climate: the psychometric quality of the prison climate questionnaire. Prison J. 100, 355–380. doi: 10.1177/0032885520916819

[ref9008] Budman S. H. Soldz S. Demby A. Davis M. Merry J. (1993). What is cohesiveness? An empirical examination. *Small Group Research* 24, 199–216. doi: 10.1177/1046496493242003

[ref8] Byrne B. M. (1998). Structural equation modeling with LISREL: Basic concepts, application and programming: Psychology Press. (Accessed March 17, 2025).

[ref9] Chen F. F. (2007). Sensitivity of goodness of fit indices to lack of measurement invariance. Struct. Equ. Model. 14, 464–504. doi: 10.1080/10705510701301834

[ref10] Chester V. McCathie J. Quinn M. Ryan L. Popple J. Loveridge C.. (2015). Clinician experiences of administering the Essen climate evaluation Schema (EssenCES) in a forensic intellectual disability service. Adv. Ment. Health Intellect. Disabil. 9, 70–78. doi: 10.1108/AMHID-06-2014-0024

[ref11] Cheung G. W. Cooper-Thomas H. D. Lau R. S. Wang L. C. (2023). Reporting reliability, convergent and discriminant validity with structural equation modeling: a review and best-practice recommendations. Asia Pac. J. Manag. 41, 745–783. doi: 10.1007/s10490-023-09871-y

[ref12] Cheung G. W. Rensvold R. B. (2002). Evaluating goodness-of-fit indexes for testing measurement invariance. Struct. Equ. Model. 9, 233–255. doi: 10.1207/S15328007SEM0902_5

[ref13] Cunha O. Castro Rodrigues A. D. Caridade S. Dias A. R. Almeida T. C. Cruz A. R.. (2023). The impact of imprisonment on individuals’ mental health and society reintegration: study protocol. BMC Psychol. 11:215. doi: 10.1186/s40359-023-01252-w, PMID: 37491401 PMC10369709

[ref14] Day A. Casey S. Vess J. Huisy G. (2012). Assessing the therapeutic climate of prisons. Crim. Just. Behav. 39, 156–168. doi: 10.1177/0093854811430476

[ref9007] de Vogel V. de Ruiter C. (2004). Differences between clinicians and researchers in assessing risk of violence in forensic psychiatric patients. Journal of Forensic Psychiatry & Psychology 15, 145–164. doi: 10.1080/14788940410001655916

[ref15] Dickens G. L. Suesse M. Snyman P. Picchioni M. (2014). Associa tions between ward climate and patient characteristics in a secure forensic mental health service. J. Forensic Psychiatry Psychol. 25, 195–211. doi: 10.1080/14789949.2014.903505

[ref16] Efkemann S. A. Bernard J. Kalagi J. Otte I. Ueberberg B. Assion H.-J.. (2019). Ward atmosphere and patient satisfaction in psychiatric hospitals with different ward settings and door policies. Results from a mixed methods study. Front. Psych. 10:576. doi: 10.3389/fpsyt.2019.00576, PMID: 31543830 PMC6728825

[ref17] Eltink E. M. A. Roest J. J. Van der Helm G. H. P. Heynen E. J. E. Kuiper C. H. Z. (2024). Safety first! Residential group climate and antisocial behavior: a multilevel meta-analysis. Int. J. Offender Ther. Comp. Criminol.:0306624X241252052. doi: 10.1177/0306624X241252052 PMC12287560 38855815

[ref18] Fornell C. Larcker D. F. (1981). Evaluating structural equation models with unobservable variables and measurement error. J. Mark. Res. 18, 39–50. doi: 10.2307/3151312

[ref19] Fox J. Weisberg S. (2019). An R companion to applied regression. 3rd Edn. Thousand Oaks, California: SAGE Publications, Inc.

[ref20] Howells K. Tonkin M. Milburn C. Lewis J. Draycot S. Cordwell J.. (2009). The essences measure of social climate: a preliminary validation and normative data in UK high secure hospital settings. Crim. Behav. Ment. Health 19, 308–320. doi: 10.1002/cbm.745, PMID: 19823989

[ref21] Hu L. Bentler P. M. (1999). Cutoff criteria for fit indexes in covariance structure analysis: conventional criteria versus new alternatives. Struct. Equ. Model. 6, 1–55. doi: 10.1080/10705519909540118

[ref22] James L. R. Demaree R. G. Wolf G. (1984). Estimating within-group interrater reliability with and without response bias. J. Appl. Psychol. 69, 85–98. doi: 10.1037/0021-9010.69.1.85

[ref23] Jorgensen T. D. Pornprasertmanit S. Schoemann A. M. Rosseel Y. (2022) semTools: Useful tools for structural equation modeling. R package version 0.5–6. Available online at: https://CRAN.R-project.org/package=semTools (Accessed March 17, 2025).

[ref9001] Krug E. G. Mercy J. A. Dahlberg L. L. Zwi A. B. (2002). The world report on violence and health. The lancet 360, 1083–1088. doi: 10.1016/S0140-6736(02)11133-0 12384003

[ref24] Kuznetsova A. Brockhoff P. B. Christensen R. H. B. (2017). LmerTest package: tests in linear mixed effects models. J. Stat. Softw. 82, 1–26. doi: 10.18637/jss.v082.i13

[ref25] Long C. G. Anagnostakis K. Fox E. Silaule P. Somers J. West R.. (2010). Social climate along the pathway of care in women’s secure mental health service: variation with level of security, patient motivation, therapeutic alliance and level of disturbance. Crim. Behav. Ment. Health 21, 202–214. doi: 10.1002/cbm.791, PMID: 21706527

[ref9009] Luborsky L. McLellan A. T. Woody G. E. O’Brien C. P. Auerbach A. (1985). Therapist success and its determinants. Archives of general psychiatry 42, 602–611. doi: 10.1001/archpsyc.1985.01790290084010 4004503

[ref26] Lüdecke D. (2024) sjPlot: Data Visualization for Statistics in Social Science. R package version 2.8.17. Available online at: https://CRAN.R-project.org/package=sjPlot (Accessed March 17, 2025).

[ref9010] Martin D. J. Garske J. P. Davis M. K. (2000). Relation of the therapeutic alliance with outcome and other variables: a meta-analytic review. J Consult Clin Psychol. 68:438, PMID: 10883561

[ref27] Meade A. W. Bauer D. J. (2007). Power and precision in confirmatory factor analytic tests of measurement invariance. Struct. Equ. Model. 14, 611–635. doi: 10.1080/10705510701575461

[ref28] Milsom S. A. Freestone M. Duller R. Bouman M. Taylor C. (2014). Factor structure of the Essen climate evaluation Schema measure of social climate in a UK medium-security setting. Crim. Behav. Ment. Health 24, 86–99. doi: 10.1002/cbm.1878, PMID: 23996523

[ref9002] Moos R. H. (1975). Evaluating correctional and community settings: Wiley-Interscience.

[ref29] Moos R. H. (1968). The assessment of the social climates of correctional institutions. J. Res. Crime Delinq. 5, 174–188. doi: 10.1177/002242786800500207

[ref9003] Moos R. H. (1974). Community oriented programs environment scale manual: Consulting Psychologists Press.

[ref9005] Moos R. H. (1996). Understanding environments: The key to improving social processes and program outcomes. American Journal of Community Psychology 24, 193–201. doi: 10.1007/BF02511887 8712186

[ref9004] Moos R. H. Cronkite R. C. Finney J. W. (1990). Health and daily living form manual. Center for Health Care Evaluation: Stanford University Medical Center.

[ref9006] Moos R. H. Houts P. S. (1968). Assessment of the social atmospheres of psychiatric wards. J Abnorm Psychol. 73:595. doi: 10.1037/h0026600 5717365

[ref9011] Rapoport R. (1960). The family and psychiatric treatment. Psych. 23, 53–62. doi: 10.1080/00332747.1960.11023202 14436271

[ref30] R Core Team. (2024). R: A Language and Environment for Statistical Computing. R Foundation for Statistical Computing, Vienna, Austria. Available online at: https://www.R-project.org/ (Accessed March 17, 2025).

[ref31] Reading L. Ross G. E. (2020). Comparing social climate across therapeutically distinct prison wings. J. Forensic Pract. 22, 185–197. doi: 10.1108/JFP-04-2020-0017

[ref32] Revelle W. (2024). Psych: procedures for psychological, psychometric, and personality research. Northwestern University, Evanston, Illinois. R package version 2.4.6. Available online at: https://CRAN.R-project.org/package=psych (Accessed March 17, 2025).

[ref33] Richardson J. Zini V. (2021). Are prison-based therapeutic communities effective? Challenges and considerations. Int. J. Prison. Health 17, 42–53. doi: 10.1108/IJPH-07-2020-0048, PMID: 33634656

[ref34] Robinson J. E. Craig L. (2019). Social climate and aggression in IDD services [social climate and aggression]. J. Intellect. Disabil. Offending Behav. 10, 8–18. doi: 10.1108/JIDOB-11-2018-0013

[ref35] Robinson J. Craig L. A. Tonkin M. (2018). Perceptions of social climate and aggressive behavior in forensic services: a systematic review. Trauma Violence Abuse 19, 391–405. doi: 10.1177/1524838016663936, PMID: 27519992

[ref9014] Røssberg J. I. Eiring Ø. Friis S. (2004). Work environment and job satisfaction: A psychometric evaluation of the Working Environment Scale-10. Soc Psychiatry Psychiatr Epidemiol 39, 576–580. doi: 10.1007/s00127-004-0791-z 15243696

[ref36] Ross M. W. Liebling A. Tait S. (2011). The relationships of prison climate to health service in correctional environments: inmate health care measurement, satisfaction and access in prisons. Howard J. Crim. Justice. 50, 262–274. doi: 10.1111/j.1468-2311.2011.00658.x

[ref37] Rosseel Y. (2012). Lavaan: an R package for structural equation modeling. J. Stat. Softw. 48, 1–36. doi: 10.18637/jss.v048.i02

[ref38] Schalast N. Groenewald I. (2009). “Ein kurzfragebogen zur einschätzung des sozialen klimas im strafvollzug” in Drogen, sucht, kriminalität. ed. Haller J. (Mönchengladbach, Germany: Forum), 329–352.

[ref39] Schalast N. Laan J. M. (2017). Measuring social climate in German prisons using the Essen climate evaluation schema. Prison J. 97, 166–180. doi: 10.1177/0032885517692792

[ref40] Schalast N. Redies M. Collins M. Stacey J. Howells K. (2008). EssenCES, a short questionnaire for assessing the social climate of forensic psychiatric wards. Crim. Behav. Ment. Health 18, 49–58. doi: 10.1002/cbm.677, PMID: 18229876

[ref42] Schalast N. Tonkin M. (Eds.) (2016). The Essen climate evaluation schema–EssenCES: a manual and more. Göttingen, Lower Saxony, Germany: Hogrefe Publishing.

[ref43] Siess J. Schalast N. (2017). Psychometric properties of the Essen climate evaluation Schema (EssenCES) in a sample of general psychiatric wards. Arch. Psychiatr. Nurs. 31, 582–587. doi: 10.1016/j.apnu.2017.08.001, PMID: 29179825

[ref44] Tomlin J. Tonkin M. (2023). The essences measure of ward atmosphere: mokken scaling, confirmatory factor analysis, and investigating patient-level characteristics. Int. J. Forensic Ment. Health 22, 199–209. doi: 10.1080/14999013.2022.2134946

[ref45] Tonkin M. (2016). A review of questionnaire measures for assessing the social climate in prisons and forensic psychiatric hospitals. Int. J. Offender Ther. Comp. Criminol. 60, 1376–1405. doi: 10.1177/0306624X15578834, PMID: 25850103

[ref46] Tonkin M. Howells K. Ferguson E. Clark A. Newberry M. Schalast N. (2012). Lost in translation? Psychometric properties and construct validity of the English Essen climate evaluation Schema (EssenCES) social climate questionnaire. Psychol. Assess. 24, 573–580. doi: 10.1037/a0026267, PMID: 22082034

[ref47] Turhan A. Roest J. J. Delforterie M. J. Van der Helm G. H. P. Neimeijer E. G. Didden R. (2024). Psychometric analysis of the group climate inventory—revised in adults with mild intellectual disability or borderline intellectual functioning in a secure residential facility. J. Appl. Res. Intellect. Disabil. 37:e13183. doi: 10.1111/jar.13183, PMID: 38043530

[ref48] Van der Helm G. H. P. Roest J. J. Dekker A. L. van Miert V. S. L. Kuiper C. H. Stams G. J. J. (2024). Group climate in residential youth care: development and validation of the group climate instrument—revised. Int. J. Offender Ther. Comp. Criminol.:0306624X231219984. doi: 10.1177/0306624X231219984 38229466

[ref49] Wickham H. (2016). ggplot2: Elegant graphics for data analysis. New York: Springer-Verlag.

[ref50] Wickham H. Averick M. Bryan J. Chang W. McGowan L. D. François R.. (2019). Welcome to the tidyverse. J. Open Source Softw. 4:1686. doi: 10.21105/joss.01686

[ref51] Wickham H. Miller E. Smith D. (2023). Haven: import and export 'SPSS', 'Stata' and 'SAS' Files. R package version 2.5.4. Available online at: https://CRAN.R-project.org/package=haven (Accessed March 17, 2025).

[ref52] Williams L. S. Green E. L. W. Chernoff W. A. (2019). “There’s more to it than just a box check”: measuring prison climate in three correctional facilities. Int. J. Offender Ther. Comp. Criminol. 63, 1354–1383. doi: 10.1177/0306624X18821090, PMID: 30600735

[ref53] Wolf E. J. Harrington K. M. Clark S. L. Miller M. W. (2013). Sample size requirements for structural equation models: an evaluation of power, bias, and solution propriety. Educ. Psychol. Meas. 73, 913–934. doi: 10.1177/0013164413495237, PMID: 25705052 PMC4334479

[ref9013] Yalom I. D. (1985). The theory and practice of group psychotherapy. 3rd Edn. New York: Basic Books.

